# Toward Molecular Medicine in Female Infertility Management: Editorial to the Special Issue “Molecular Mechanisms of Human Oogenesis and Early Embryogenesis”

**DOI:** 10.3390/ijms222413517

**Published:** 2021-12-16

**Authors:** Jan Tesarik

**Affiliations:** MarGen Clinic, Molecular Assisted Reproduction and Genetics, Camino de Ronda 2, 18006 Granada, Spain; jtesarik@clinicamargen.com

Female infertility is the main reason for involuntary childlessness nowadays. The overall trend toward postponing parenthood toward a more advanced female age in most of the economically developed countries is certainly partly, though not exclusively, responsible for this trend. A number of age-independent factors, related to professional gametotoxic exposures and other independent environmental factors can also come into play. The impact of all these factors in each individual woman is conditioned by her genetic and epigenetic background.

Apart from the known genetic [1] and epigenetic [2] abnormalities impairing female fertility, there are a number of additional, more subtle oocyte-derived conditions that can also have an effect. This is especially the case of human embryos, because of the relatively late onset of their gene activation. In fact, in all animal species, the earliest developmental stages following fertilization are controlled by maternally inherited genetic information, stored in the form of polyadenylated messenger RNA (polyA mRNA) species, until the onset of the expression of the own embryonic genes of both paternal and maternal origin [3]. The onset of the embryonic gene expression marks the initiation of the transition from the maternal to the embryonic control of embryonic development. 

Unlike the most studied mammalian models, the maternal-to-embryonic developmental control transition occurs later in humans. The first signs of embryonic RNA synthesis (transcription) were observed in the nucleoli of four-cell human embryos [4], while extranucleolar RNA synthesis was first observed in eight-cell embryos [4], coinciding with the first signs of the expression of embryo-derived transcripts [5] and the early ultrastructural changes typical of the accomplished maternal-to-embryonic control shift [6]. However, as in other species [3], the influence of the maternally inherited and embryo-derived transcripts on further embryonic development overlap in human embryos; therefore, maternally derived transcripts can exert effects on embryonic development even after the onset of embryonic gene expression [7].

The relatively late onset of embryonic gene expression in human embryos underscores the importance of the molecular events taking place during oogenesis, so as to endow the future embryos with an appropriate stock of maternal mRNAs as well as of the molecules controlling their coordinated translation before, during, and after the embryonic genome’s activation [8]. These molecules are involved in both the degradation of maternal mRNAs and the initiation of embryonic transcription, both of which have to be finely coordinated in order to ensure optimal embryonic viability. 

The oocyte-derived epigenetic patterns controlling early embryonic development are no less important than the genetic ones [9]. In fact, there is a lot of overlap between the genetic background and epigenetic remodeling in the control of early embryogenesis, including the effects of changes in DNA methylation [10], histone modifications [11], chromatin accessibility [12], and 3D chromatin organization [13] on the expression of maternally derived transcripts and the subsequent embryonic gene activation. 

The information derived from the studies focused on the above aspects of the relationship between oogenesis and the early embryogenesis can serve as a guide for further in-depth analysis of the molecular particularities that can guide a doctor to design the optimal diagnostic and treatment strategies for each individual patient. 

A failure of implantation and early pregnancy loss, independent of embryo quality, is another possible cause of female infertility [14]. In addition to previously undetected uterine cavity abnormalities, which can now be easily detected with the use of non-invasive ultrasound-based digital reconstructions [15], immunological factors are currently the focus of attention [16]. Given the fact that immunological factors, supposedly implicated in implantation failure, were shown to be different in the uterine cavity as compared to peripheral blood [17], further high-quality molecular studies are urgently needed to resolve this issue.

Preserving genome integrity during the cleavage stage of early embryogenesis is another essential goal to be achieved by the embryo, with regard to its potential to generate multiple, distinct cell lineages [18]. A mammalian embryo’s ability to respond to damaged DNA and repair it, as well as its sensitivity to specific lesions, is still not well understood [19], and there is an urgent need to clarify this issue in order to improve the current success rates of infertility treatment techniques. The search for specific gene mutations responsible for genomic instability in human early embryonic cells [20] is one of the possible approaches. Once gene repair in the germline is proven harmless and legally accepted for human application, these data will be useful for choosing the best strategy to be employed. 

The ability of the oocyte to repair sperm-derived DNA damage represents another challenge for future research. Sperm DNA damage is known to cause genomic instability in early embryonic development [21]. However, this issue can be handled, to some extent, by the oocyte-derived repair mechanisms that are closely related to maternal age, when the wife’s own oocytes are used, or to the age of the oocyte donor in the case of recourse to donated oocytes [22]. In fact, a combination of sperm DNA damage and advanced maternal age is the worst scenario, which should be avoided to achieve the best chances of livebirth [22]. However, currently, this is merely an ultimate-recourse solution, and further studies are expected to enable childbirth for older women whose husbands show signs of excessive sperm DNA damage, without recurring to donor oocytes and thus allow the genetic information of the mother to be transmitted to the offspring. 

We still need more high-quality studies to suggest ways to improve female fertility status by increasing the quality of oocytes in women of any age. The mechanisms responsible for both the physiological [23] and premature [24] ovarian aging appear to be similar and closely related to a defective mitochondrial function leading to oxidative stress. 

This Special Issue aims at advancing our knowledge and understanding of how to design molecular studies in both humans and animal models and to use and interpret their results to improve different aspects of oocyte quality related to the success of both natural and assisted reproduction outcomes. The data obtained from both humans and different animal models will be used to predict the risk of premature ovarian decay and the ways to prevent it, the molecular diagnostic of the existing issues, and the ways to act in each individual case, also taking into account the condition of the male partner. The synthesis of these analyses will serve to determine (1) the need for taking therapeutic actions, (2) the degree of urgency of the actions to be taken, and (3) the possibility of adopting a wait-and-see approach in patients who either do not have a stable male partner or in whom immediate action is not currently necessary.

## Data Availability

The data supporting reported results can be obtained from the MARGen Clinic, Camino de Ronda 2, 18006 Granada, Spain (jtesarik@clinicamargen.com).
